# On the etiology of internalizing and externalizing problem behavior: A twin-family study

**DOI:** 10.1371/journal.pone.0230626

**Published:** 2020-03-23

**Authors:** Amelie Nikstat, Rainer Riemann

**Affiliations:** Department of Psychology, Bielefeld University, Bielefeld, Germany; McMaster University, CANADA

## Abstract

Internalizing and externalizing behavior problems are established risk factors for many unpleasant outcomes and psychopathology in adulthood, and understanding the interplay between genes and environment is important for deducing implications for therapeutic interventions. Among genetic studies on internalizing and externalizing problem behavior, the heritability estimates differ widely. Most research only uses twin data and other-reports, and therefore certain limitations are inevitable. Our study is the first to investigate genetic and environmental influences on problem behavior using a Nuclear Twin Family Design and self-reports, in order to address these limitations. Internalizing and externalizing problem behavior of 3,087 twin pairs (age 11–23), a sibling, and their parents were analyzed with structural equation modeling to estimate heritability separately for each of three twin birth cohorts. Genetic influences account for about one-third of the variance for both internalizing and externalizing. Shared environmental influences were only found for internalizing, and through the advantages of considering data from the whole twin family, firstly could be identified as solely twin-specific. Our findings could contribute to a better understanding of the gap between heritability based on twin studies and DNA-based heritability (‘missing heritability problem’): Results indicate that heritability estimates gained via classic twin design and other-reports are slightly overestimated and therefore environmental influences, in general, are more important than previous research suggests. Simultaneously, we showed that family-specific environment either contributes to behavior problems only on an individual level, or that it has a lesser influence than originally thought.

## Introduction

*Internalizing* and *Externalizing* are two broad categories of behavioral problems: Whereas internalizing problem behavior (INT) is focused on the own self (e.g., withdrawal, anxiety, depression, emotional problems), externalizing problem behavior (EXT) particularly occurs in interaction with the social environment (e.g., aggression, impulsivity, deviance, hyperactivity). The terms were introduced in 1966 and derived by factor analysis of children’s psychiatric symptoms [1]. Understanding the etiology of INT and EXT in childhood and adolescence is of great importance because of the high prevalence [2, 3] and the association with numerous unfavorable development outcomes, including poor academic performance, antisocial behavior, delinquency, peer problems, and poor mental health [4–7]. In the light of these strong associations, it is no surprise that INT and EXT are assumed to reflect the underlying broader dimensions of the common, categorical diagnosis system of mental and personality disorders and to account for covariation among symptoms of different disorders [8, 9]. Given this high significance of INT and EXT, answering the question what factors are responsible for their development and maintenance is crucial. Therefore studying the pattern of genetic and environmental influences has important implications for the optimal timing and nature of interventions.

Behavioral genetic research on INT and EXT yielded heterogeneous estimates of genetic and environmental influences. Heritability estimates varied between about .30 and .75 [10–13], whereby EXT, on average, shows greater heritability than INT. Like most traits [14], genetic influences on INT and EXT are typically smaller in early childhood and increase with age [15]. Environmental influences not shared by family members account for a substantial part of the variance, whereas environmental influences shared by siblings often are negligible [16]. Nevertheless, Burt [17] concluded that shared environmental influences may actually play a bigger role due to methodological and power issues in many studies, but empirical work which addresses both of these limitations is still missing. Highly stable genetic influences, as well as smaller genetic and environmental innovations, were observed across ages, suggesting that early emerging and enduring biological differences reflect the phenotypes, and that genetic influences unfold across childhood and adolescence [18]. The transition between adolescence and adulthood is not well explored because most studies have focused either on younger children or adults. Against this background, research with the aim to investigate the heritability of INT and EXT has to consider different stages of development as a source of variance.

One of the reasons for varying heritability estimates in previous studies are differences in methodology. Older studies tend to estimate heritability from twin correlations, based on some rules of thumb, whereas newer work uses many different types of structural equation modeling. This methodology allows for a more precise estimation, but is in fact also based on twin correlations only. In the following, we briefly explain the basics of the commonly used classical twin method (CTM), which relies on data from twins reared together. Monozygotic (MZ) twins share 100% of their segregating genes, whereas dizygotic (DZ) twins share, on average, 50% of their genes. Making use of these known differences, the variance of a phenotype can be partitioned into three of four possible components: additive (a^2^) and non-additive (i^2^) genetic variance, and shared (c^2^) and non-shared (e^2^) environmental variance. a^2^ is the effect of individual genes summed over gen-loci, i^2^ describes gene-to-gene interactive effects at the same or across multiple loci (referred to as epistasis). c^2^ is that part of the environment that is common for siblings reared together and makes them similar to each other, whereas e^2^ is unique to every sibling, making them less similar. e^2^ includes measurement error (which similarly acts to reduce sibling correlations). i^2^ and c^2^ cannot be estimated simultaneously in the CTM because these parameters are estimated using the same information.

To address the limitations of the CTM, the inclusion of data from parents and siblings into twin studies (nuclear twin family design, NTFD) has been suggested [19, 20]. The NTFD can disentangle different sources of c^2^, namely environmental sources which are shared by children and mothers (m^2^) and fathers (f^2^), sources that affect siblings (cs^2^), and sources that are shared only by the twins (ct^2^). Using the NTFD, we can estimate i^2^ in the presence of most c^2^-components (but not cs^2^). Besides, in the CTM, it is not possible to estimate effects of assortative mating, which—at present—leads to an underestimation of a^2^ and an overestimation of c^2^. Finally, the NTFD also can model passive gene-environment correlation, meaning that children’s genetic endowment is correlated with their family environment.

Another reason for varying estimates across studies could be that they are based on different groups of informants (mostly parents or teachers). For example, heritability for different types of problem behavior is significantly higher when twins are rated by the same teacher than rated by different teachers [12, 21]; only small and non-significant environmental influences for teacher ratings were found, in contrast to significant non-shared environmental influences for parental ratings [22]. In addition, the correlations among different groups of raters are only moderate (in most cases *r* ≤ .30) and decrease with the age of the child [23–25]. Given these differences, self-reports provide a valuable perspective which supplements parents’ and teachers’ perspectives, particularly considering that children may behave differently in different environments (such as the presence of teachers and parents), and are not completely open about all their non-observable, internal processes. INT symptoms are particularly elusive, whereas EXT is more easily observable from the outside and also more likely to cause social problems. For example, Comer & Kendall [26] found stronger parent-child agreement for observable symptoms than for unobservable symptoms and weaker agreement for school-based than for non-school-based symptoms. Sourander, Helstelä & Helenius [27] showed that INT among adolescent girls is often unrecognized by adults. Taken together, the most commonly suggested reason for disagreement between informants is the tendency for children’s INT and EXT symptoms to be differentially observable depending on the situation [23, 25, 26, 28].

In general, genetic and shared environmental factors are estimated higher for other-reported data, whereas non-shared environment seems to play a bigger role for self-reported problem behavior [13, 17, 29, 30]. To our knowledge, only Scourfield et al. [13] compared heritability estimates based on self-reports in detail with parent and teacher reports. In their study, heritability of self-reported conduct problems was significantly lower (35% of the variance) than heritability estimated from parent- and teacher-reports (54% and 77%, respectively), whereas no significant effects of shared environment were found at all. Nevertheless, in their multivariate analyses, the variance shared by all rating methods (true score variance) was entirely due to genetic influences (62% additive genetic effects and 38% non-additive genetic effects). Furthermore, comparability between age groups is crucial for the NTFD, where data from the whole family (in which age differs widely, particularly between parents and children) is considered. It is only given when the same instrument is used for all family members. Self-report is the most widely used method for personality related constructs in adolescence and adulthood, and data from multiple sources is often not available. Although it is sometimes suspected that adolescents’ self-reports of problem behavior are biased through the impact of social desirability, several studies found that in non-clinical samples, adolescents report frequencies of problem behaviors that are higher than those based on ratings by parents or teachers, that is, they report more undesirable behaviors [13, 31–33]. Thornberry & Krohn [34] found that generally adolescents answer such questions truthfully and concluded that self-report data on problem behavior “appear acceptably valid and reliable for most research purposes” (p. 33). Additionally, recent research has shown evidence for the predictive power of children’s self-reports [35]. Thus, since self-reports could provide an interesting new perspective on heritability of INT and EXT and as there is evidence for their validity, we base our current study on self-reported INT and EXT. Based on the findings for self-reported heritability compared to estimates gained from teacher- or parent-reports [13], we expect heritability to be slightly lower than in previous, other-report based work.

In summary, discrepant findings for the heritability of INT and EXT are likely to be the result of different sample characteristics, such as age, different rater types and measurement strategies, and different and limited methodological approaches. The current study uses a nuclear twin family design, taking into account different age groups, and the more comprehensive view of self-reports, in order to provide a more accurate view of the different variance components of INT and EXT, and the resulting practical implications. Although our study is largely explorative, we have the following expectations: (1) Heritability is slightly higher for EXT than for INT [10, 12, 17]. (2) Heritability increases across age groups, whereas environmental influences decrease [14, 15]. (3) Because it is suggested that earlier studies found no shared environmental influences on behavioral problems only due to methodological and power issues [17], we expect to find at least small shared environmental effects with the NTFD. (4) In line with the meta-analysis of Burt [17], we expect no or only small non-additive genetic effects. (5) Overall, heritability is slightly lower than in most previous work due to the use of self-reports [13].

## Method

### Sample and procedure

Participants included 3,087 twin families from the TwinLife project, which is an ongoing genetically informative, cross-sequential study of social inequalities [36]. The study received ethical approval from the German Psychological Society (Deutsche Gesellschaft für Psychologie; protocol number: RR 11.2009), therefore complying with the ethical standards of the 1964 Helsinki declaration and its later amendments. Informed consent was obtained and recorded from all individual participants during the household interviews. A parent provided parental consent for adolescent participation (see S1 File for additional information on participant consent). The sample is representative of the German population and covers the full distributions for core social inequality indicators like educational status, occupational status and income [37]. The TwinLife study provides data of four birth cohorts of same-sex MZ and DZ twin pairs, their parents, one sibling of the twins and partners of the older twins. The first assessment wave took place in 2014–2016 and was split into two half-waves of data collection. For twins of the youngest cohort (aged 5 at the time of assessment) and siblings under the age of 10, no self-reports of INT and EXT were available, therefore they were excluded from our analyses. Using the whole first survey wave, data of three birth cohorts of same-sex twin pairs (age 11, 17, and 23 at the point of data collection, therefore in the following named C11, C17 and C23), one full sibling, and their biological mothers and fathers were included in the current analyses. Characteristics of the final sample are shown in Table 1.

**Table 1:** Sample characteristics.

	N	Age M (SD)	Sex male (%)	Zygosity MZ (%)
**total**				
Twin pairs	3087	16.9 (4.9)	1364 (44.2)	1443 (46.8)
Sibling	1305	19.7 (5.8)	674 (51.6)	
Mother	3002	47.6 (6.1)		
Father	2400	50.4 (6.4)		
**C11**				
Twin pairs	1043	11.0 (0.3)	500 (47.9)	421 (40.4)
Sibling	380	14.9 (2.6)	194 (51.1)	
Mother	1034	42.9 (4.9)		
Father	880	46.4 (5.6)		
**C17**				
Twin pairs	1061	17.0 (0.3)	453 (42.7)	498 (47.0)
Sibling	474	18.9 (4.4)	259 (54.6)	
Mother	1023	47.7 (4.6)		
Father	819	50.5 (5.1)		
**C23**				
Twin pairs	983	23.1 (0.8)	411 (41.8)	524 (53.4)
Sibling	451	24.6 (5.0)	221 (49.0)	
Mother	945	52.6 (4.6)		
Father	701	55.3 (5.3)		

C, birth cohort; MZ, monozygotic twins.

### Measures

#### Zygosity

Zygosity was determined with the *Zygosity Questionnaire for Young Twins* [38] in C11, and with the *Self Report Zygosity Questionnaire* [39] in C17 and C23. Correct classification rates of 97% for parent- and 92% for self-reports were established with DNA-based zygosity (N = 328 twin pairs) as criteria (if DNA-based zygosity diagnoses were available, they were used; see Lenau et al. [40] for more details).

#### INT and EXT

We collected computer-assessed self-reports on four of the five subscales of the German translation of the *Strengths & Difficulties Questionnaire* (SDQ) [41], a short instrument for measuring psychosocial problems in children and adolescents that was primarily developed as a screening instrument for population-based samples [42]. The subscales *anxiety* and *peer problems* represent INT, whereas the subscales *hyperactivity* and *conduct* reflect EXT. Each subscale consists of five items that are answered on a 3-point Likert-type scale, ranging from 0 = *not true* to 2 = *certainly true*. Recently, configural measurement invariance between the self- and parent-report version was shown [43], indicating that both versions measure the same constructs, and thus, are comparable on a phenotypic level. Based on a systematic review of 54 studies reporting on the factor structure of the SDQ, and their own analyses, Caci, Morin, and Tran [44] conclude that altogether, a bifactor model with an additional representation of the broader dimensions of INT and EXT is a superior representation of different INT and EXT problems compared to models which only depict the original factor structure based on the problem subscales. Relatedly, Goodman, Lamping, and Ploubidis [45] suggested using the broader INT and EXT scales for analyses in low-risk samples while retaining the original four subscales when screening for disorder. Based on these elaborations, we did not focus on the four problem subscales, but used the broader dimensions of INT and EXT as an appropriate representation of problem behavior in our non-clinical sample.

Participants between 10 and 17 years were given the child version, and older participants were given the adult version (see http://www.sdqinfo.com for both versions). The SDQ correlates strongly with related scales like the Youth Self-Report and the Child Behavior Checklist and shows good discriminant and concurrent validity [46–48]. For the SDQ, normative data is available (see also http://www.sdqinfo.com). In line with previously reported reliabilities for the SDQ [43, 49–51], in our sample, McDonald’s ω was .70 for INT and .63 for EXT (Cronbach’s α was .69 and .62) across all participants. One item was not assessed in the adult sample (“I am constantly fidgeting or squirming”). Means and standard deviations for the present sample were also in line with expectations (see Table 2). Configural measurement invariance across cohorts was given for both, INT and EXT, whereas metric measurement invariance was only given for EXT for the cohorts C17 and C23 (see S1 Table).

**Table 2:** Descriptive statistics for internalizing and externalizing.

	Internalizing		Externalizing	
	male	female	male	female
Twins C11	.44 (.30)	.48 (.31)	.57 (.31)	.55 (.32)
Twins C17	.39 (.28)	.59 (.31)	.45 (.29)	.44 (.28)
Twins C23	.41 (.30)	.53 (.32)	.42 (.27)	.38 (.27)
Siblings	.39 (.29)	.53 (.31)	.49 (.30)	.44 (.29)
Parents	.36 (.27)	.44 (.30)	.33 (.23)	.34 (.22)

C, birth cohort; values given are mean (standard deviation).

## Analyses and results

We used SPSS 25 [52], AMOS 24 [53], and JASP 0.9 [54] for our analyses. Descriptive statistics were calculated based on the raw scores. As a result of having a representative, non-clinical sample, our data was slightly right-skewed. Therefore, we log-transformed the INT and EXT scales for correlation analysis and structural equation modeling to obtain more adequate symmetry. For correlation analysis and structural equation modeling, also linear age and sex effects were controlled using standardized residuals. Adjusting for age and sex in twin studies is an established practice because age and sex differences affect covariance among family members, and failing to correct for these differences can result in biased estimates of genetic and environmental variance contributions [55]. In line with previous work [56], across all participants, females scored significantly higher on INT (β = .11, *p* < .001), but significant lower on EXT (β = -.02, *p* < .001), whereas a higher age was significantly associated with lower scores on both scales (INT: β = -.002, *p* < .001; EXT: β = -.004, *p* < .001).

### SDQ kin correlations

The correlations among all family members are shown in Table 3. For INT, all kin correlations were significant. Correlations between MZ twins were clearly higher than correlations between DZ twins, suggesting genetic influences (a^2^). DZ correlations for INT were higher than half of the MZ correlations, suggesting shared environmental influences (ct^2^ and/or cs^2^). All twin–sibling and parent–child correlations were lower than the DZ twin correlation, suggesting twin-specific environmental influences (ct^2^). The correlation between parents indicated weak phenotypic assortative mating (μ). For EXT, there was a similar pattern, although all correlations were lower than for INT, and the correlations between sibling and both parents were non-significant. The correlation between the DZ twins was even less than half of the MZ correlation, pointing to non-additive genetic effects (i^2^). In addition, the DZ correlation was only slightly higher than the twin–sibling and parent–child correlations, indicating that shared environmental influences do not play a significant role for EXT.

**Table 3:** Kin correlations.

	Internalizing		Externalizing	
	*r*	95% CI	*r*	95% CI
MZ T1–T2	.44*	.399–.482	.37*	.320–.410
DZ T1–T2	.29*	.240–.330	.13*	.085–.180
T1–S	.18*	.112–.237	.11*	.044–.177
T1–M	.16*	.126–.198	.13*	.091–.164
T1–F	.15*	.100–.192	.11*	.064–.157
T2–S	.19*	.127–.252	.12*	.053–.181
T2–M	.16*	.122–.194	.11*	.077–.150
T2–F	.10*	.054–.147	.09*	.042–.135
S–M	.17*	.107–.237	.07	-.002–.131
S–F	.18*	.096–.253	.06	-.018–.143
M–F	.14*	.088–.188	.12*	.070–.168

MZ, monozygotic twins; DZ, dizygotic twins; T1, first-born twin; T2, second-born twin; S, sibling; M, mother; F, father;

* *p* < .001

### NTFD

**Fig 1:**
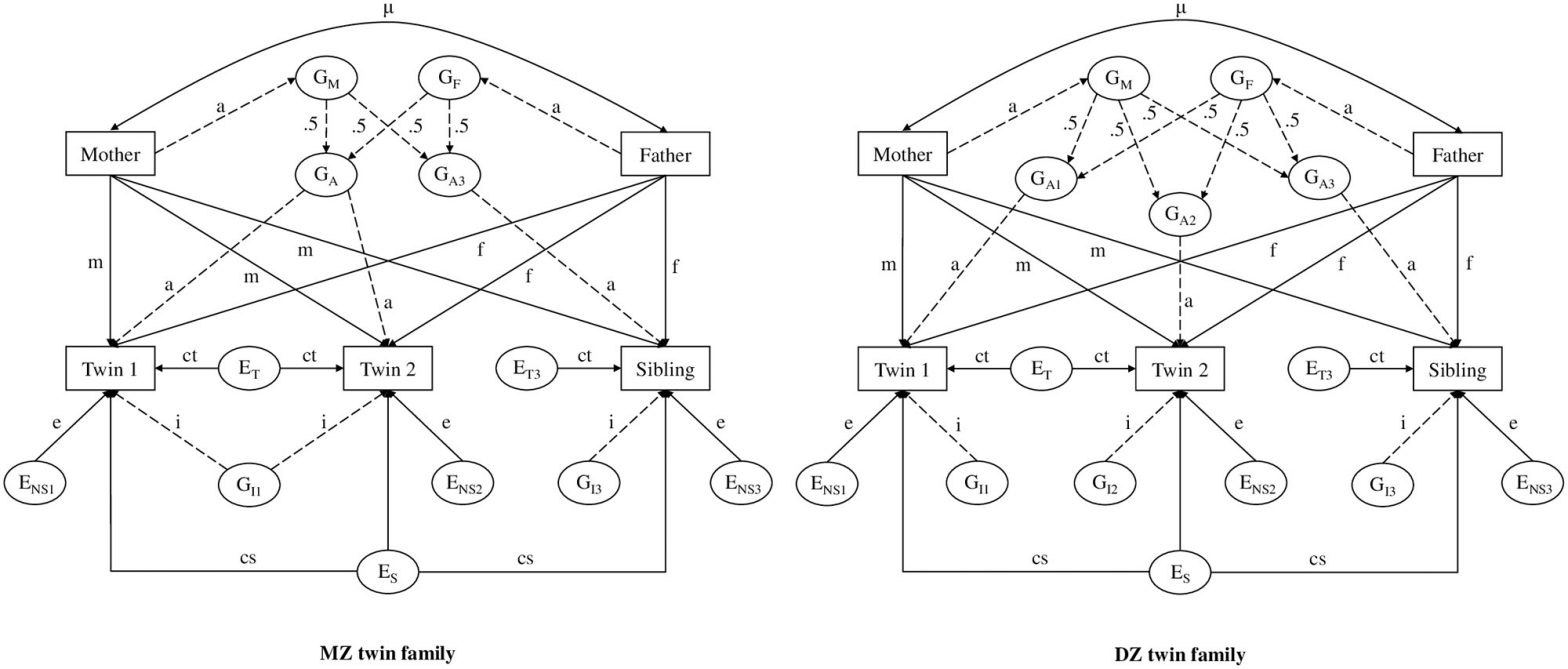
Nuclear twin family design model. G, genetic factors; E, environmental factors; dotted lines, genetic paths; a, additive genetic effects; i, non-additive genetic effects; m, environmental transmission from mother to offspring; f, environmental transmission from father to offspring; cs, environmental effects shared by siblings; ct, environmental influences shared by twins; e, unique environmental influences (including measurement error); μ, parent’s phenotypic similarity.

We estimated NTFD parameters (see Fig 1) with Full Information Maximum Likelihood, as described in Bleidorn, Hufer, Kandler, Hopwood, and Riemann [57]. We ran a multi-group analysis with birth cohort and zygosity as grouping variables. The models for INT and EXT were estimated separately. To obtain the best-fitting and parsimonious models, we tested a series of models, beginning with the full model and moving to nested, more parsimonious models. To test if a more parsimonious model fitted the data significantly worse, we used the chi square difference test [58]. If there was no significant difference, the more parsimonious model was chosen.

We fitted the full NTFD to data from C11, C17, and C23 simultaneously. As i and cs cannot be estimated simultaneously, we tested i = 0 and cs = 0 for all cohorts as two alternative baseline models, whereas all other parameters were set free, and allowed to differ between groups. For both INT and EXT, the cs = 0 model showed a better fit and was set as baseline, although for INT, the difference between both models was very small. In the second step, we set each parameter equal across cohorts to test invariance between cohorts. Whereas for INT, the model fit remained roughly on the same level when equating parameters across cohorts, for EXT, the model fit was significantly worse, indicating significant differences in etiology between cohorts. In the third step, we ran a series of models, in which we tested whether non-significant model parameters in the baseline model could be fixed step-by-step to zero without a significant decline in model fit. The most parsimonious solution, which showed no decrease in model fit compared to the baseline model, was chosen as a final model and is reported here (see Table 4 for fit statistics and S2 Table for model parameters and variance components).

**Table 4:** Model fit.

Model	χ^2^ / df (*p*)	CFI	RMSEA	AIC	χ^2^_diff_ / df_diff_ (*p*_*diff*_)
*Internalizing*					
**M1.1** (baseline) cs = 0	68,25 / 69 (.503)	>.999	<.001	170,254	
**M1.2** i = 0	68,68 / 69 (.488)	>.999	<.001	170,682	
**M2** cs = 0, o. eq.	83,14 / 83 (.510)	>.999	.001	157,144	14,89 / 14 (.386)
**M3 (final model)** cs = i = m = f = 0; o. eq	85,00 / 86 (.468)	>.999	<.001	153,002	16,75 / 17 (.472)
*Externalizing*					
**M1.1** (baseline) cs = 0	66,54 / 69 (.562)	>.999	<.001	168,536	
**M1.2** i = 0	74,92 / 69 (.292)	.976	.005	176,92	
**M2** cs = 0, o. eq.	95,88 / 88 (.158)	.960	.007	169,883	29,35 / 14 (.009)
**M3 (final model)** cs = m = f = ct = 0	69,00 / 78 (.757)	>.999	<.001	152,996	2,46 / 9 (.982)

M, Model; C, birth cohort; cs, environmental effects shared by siblings; i, non-additive genetic effects; m, environmental transmission from mother to offspring; f, environmental transmission from father to offspring; ct, environmental influences shared by twins; o. eq., all other Parameters were set equal across cohorts

For INT (no cohort differences), non-additive genetic variance (i) and direct influences from parents to offspring (m, f) could be fixed to zero. The remaining reduced model indicated the presence of significant additive genetic (a), non-shared environmental (e) and twin specific environmental effects (ct), and a significant correlation between the parents (μ). For EXT (significant cohort differences), sibling-specific shared environment variance and direct influences from parent to offspring could be fixed to zero, too, but there was significant non-additive genetic variance (i), whenever twin specific environmental paths could be dropped. The parsimonious model for EXT contained significant parameters for additive and non-additive genetic variance, non-shared environmental variance, and a significant correlation between the parents (see Table 5 for standardized variance components of the final models). For EXT the confidence intervals of all parameters overlap between the cohorts (with the exception of e^2^, for which C11 and C17 barely did not overlap). For this reason, we tested whether the significant decline in model fit, when setting all parameters equal across cohorts, could be due to variance differences between the cohorts (Phenotypic variance decreases slightly from C11 to C23, cf. standard deviations in Table 2). We standardized the variances for each cohort separately and run our models with this data. Overall, the baseline models and path coefficients remained the same, while model fit increases slightly. For INT, there was no difference concerning model reduction. But for EXT, the model were all parameters were set equal across cohorts showed no significant decline in model fit compared to the baseline model anymore. Fit statistics and standardized variance components of the alternative models are provided in S3 Table. Because this procedure was purely explorative, we do not report the resulting solution as our final model. However, from these analyses we conclude that the cohort differences in our final model are due to differences in the variance rather than the size of parameter estimates.

**Table 5:** Standardized variance components (final models).

	a^2^	i^2^	ct^2^	e^2^
Internalizing	.32 [.302–.330]		.12 [.096–.135]	.57 [.562–.576]
Externalizing C11	.21 [.184–.234]	.13 [.090–.179]		.66 [.644–.672]
Externalizing C17	.20 [.178–.229]	.18 [.136–.217]		.62 [.607–.635]
Externalizing C23	.25 [.223–.278]	.11 [.072–.162]		.63 [.621–.648]

C, birth cohort; [], 95% confidence interval; a, additive genetic effects; i, non-additive genetic effects; ct, twin-specific shared environmental effects; e, non-shared environmental effects (including measurement error)

Summed up, as expected from the patterns of kin correlations, there was significant and substantial genetic variance for both INT and EXT. Genetic influences account for 32% of the variance for INT, and between 34% and 38% of the variance for EXT. Non-additive genetic effects were significant only for EXT. For both scales, the largest variance component was non-shared environmental variance. As suggested by the kin correlations, a smaller proportion of variance was due to twin-specific shared environmental effects for INT, but not for EXT. Both models suggested that assortative mating is present, but neither cultural transmission nor passive gene–environment covariance had substantial effects. Although they do not seem to be significant, parameter differences between the cohorts in the EXT model showed an unexpected pattern: The highest heritability and the lowest non-shared-environment effect were found for C17, followed by the oldest cohort (C23). However, as expected, the lowest heritability and the highest environmental effect were found for the youngest cohort (C11).

## Discussion

The aim of this report was to provide a more accurate view of the different variance components of INT and EXT by using an NTFD. We collected self-reports of three birth cohorts of twins and their families in a population-based, normative sample. Our expectations were only met in part. In line with previous research and supporting our hypotheses, genetic influences accounted for approximately one-third of the variance for both INT and EXT, with slightly higher heritability estimates for EXT. Differences between the age cohorts were only found for EXT and were not due to differences in parameter estimates, but due to differences in variance. In line with meta-analytic findings [17], there were significant shared environmental influences on INT, but -contrary to Burt’s [17] results- not on EXT. On the other hand, we found unexpectedly high non-additive genetic effects for EXT. Finally, in line with our suggestions, heritability for both, INT and EXT was lower than in most previous work.

Besides the possibility to differentiate between various components of shared environment and to simultaneously estimate non-additive genetic effects, using an NTFD based on self-reports addresses two important limitations of prior work. CTM-based analyses are prone to overestimating genetic influences [57]. Moreover, other-reported problem behavior showed higher heritability than self-reports [17, 29], and also seemed to lead to an overestimation of genetic influences [21]. By using the NTFD and self-reports, we avoided these sources of overestimation and provided a more valid view on the heritability of INT and EXT. With about one third of the variance for INT, and slightly higher estimates for EXT, the amount of genetic influences is lower compared to most previous findings based on other-reports and the CTM (for example, Burt [17] reported an average heritability of .59 for EXT, and .51 for INT across all studies she considered in her meta-analysis). Thus, we can correct the commonly assumed extent of genetic variance of INT and EXT downwards and therefore emphasize the importance of environmental factors. Furthermore, in the CTM, assortative mating would show up as shared environmental influence. By modeling the correlation between parents in the NTFD, we were able to eliminate this bias, which leads to more confident estimates. We found no passive gene–environment correlations for INT or EXT. This is in line with the reasoning that passive gene–environment correlations are thought to dissipate throughout childhood, and to be non-existent by adulthood [17], given our sample of rather older children and adolescents.

Our results could also explain a part of the ‘missing heritability problem’, which describes the gap between heritability based on twin studies and DNA-based heritability. For most complex traits, heritability estimates based on twin studies are roughly twice as high as DNA-based heritability. For childhood problem behavior, this gap is even significantly larger. For example, in a recent study Cheesman et al. [30] found an average twin heritability of 52%, while the average DNA-based heritability was just 6%. The gap was largest for parent- and teacher-reported behavior problems, but diminished slightly for self-reports (37% twin based heritability and 5% DNA-based heritability) [30]. Considering our own results, part of the gap may be due to an overestimation of heritability in other-report-based twin studies using the CTM. Furthermore, another part of the difference is often thought to be due to non-additive genetic effects, because DNA-based analyses cannot detect non-additive genetic effects, and in the CTM they cannot be estimated in presence of shared environmental effects and therefore could contribute to the differences if remaining undetected. Because in our study we were able to estimate non-additive genetic effects in presence of most c^2^-components, this source of difference could be minimized. With a heritability of 32% for INT we could explain nearly half of the difference between twin-based and DNA-based average heritability estimates reported by Cheesman et al. [30]. For EXT this part was even larger, considering that about one third of the average genetic influence was due to non-additive genetic effects, resulting in only 22% additive genetic variance.

### Genetic and environmental influences on INT

For INT, non-shared environmental influences account for the largest part of the variance. With 12% a significant part of the variance was due to shared environmental influences. Compared to the results of Burt [17], this estimate is quite high and more in line with the average findings over different raters (12–16%) than for self-reports (8%). Furthermore, the NTFD allows distinguishing between environmental effects shared by parents and offspring, by siblings, and environmental effects that only are shared by the twins. We only found effects shared by twins, but not by other family members, suggesting that it is not the shared family surrounding that ultimately matters but rather twin-specific environments. These twin-specific environmental influences could be due to age-specific social experiences (i.e., unlike other family members, twins experience important events like divorce of parents at the same age, and thus from a similar perspective), or due to the greater extent in which they share environments (e.g., classrooms, peers) compared to other siblings. In addition, there might indeed be influences specific to twins in particular. Twins tend to attract special attention in social environments when both members of the twin pair are present, and they not only appear as individuals but also have a couple identity [59, 60]. Furthermore, if reared together, they spend more time with each other than with any other person [61]. Twin-specific influences may also reflect their special social interaction, which is often more intense between twins than among other siblings [62].

Genetic influences account for about one-third of the variance in INT and are similar across age-cohorts. Contrary to previous research, we found no increasing genetic or decreasing environmental influences across the three birth cohorts. However, this result is in line with a recent study of Patterson et al. [63], and with Bartels et al. [10, 64], who suggested that the relative importance of genetic effects decreases from age 3–7, but remains relatively stable from age 7–10. The characteristic pattern of increasing genetic and decreasing environmental influences is assumed to be a function of gene–environment interplay, i.e., increasing autonomy may result in greater freedom to choose environments consistent with the genetic predisposition. Developmental theory and evidence highlight that especially through puberty and adolescence, certain new social demands and stressors become important for the vulnerability to INT symptoms [63], which may lead to a different pattern of influence. In terms of genetic and environmental contributions on INT, both of the above could balance each other out. However, note that in our sample, the youngest cohort was already near the typical onset of puberty, where some big steps to autonomy were already taken, and puberty-specific environmental challenges might have begun to start. Considering this and results from longitudinal work—which found genetic influences to be relatively stable, whereas environmental influences to be responsible for changes—it is conceivable that the relative importance of genes and environment for INT change in earlier childhood, but remain relatively stable from the beginning of adolescence through adulthood.

### Genetic and environmental influences on EXT

In contrast to our findings for INT and the meta-analysis of Burt [17], EXT did not appear to be influenced by shared environment and showed significant non-additive genetic influences. Nevertheless, our results are quite similar to Burt’s [17] findings on attention-deficit hyperactivity problems (ADHP), which represent one facet of EXT traits thought to have a special role among within the construct. For hyperactivity, the obtained pattern is a stable finding. In our study, the EXT scale contains only subscales for ADHP and conduct problems, and the items for ADHP are very homogeneous, whereas the items for conduct problems are very heterogeneous. This leads to a relatively strong representation of ADHP in our study compared to other investigations of EXT. Thus, the exact operationalizing of EXT may play a large role and explains the similarity of our results to findings specific to ADHP. In addition, contrary to the CTM, we took assortative mating into account, and thus avoided an overestimation of c^2^ effects. However, given the small size of the biological parents’ correlation, this is only a minor correction. Furthermore, we found just small, and probably not reliable differences for the parameter estimates between the cohorts. Contrary to our expectations, no continuous increase in genetic and decrease in environmental variance was found across cohorts. Steinberg and Morris [65] argued that heritability increases up to the end of puberty, but decreases slightly afterwards, to remain on a relatively stable level in adulthood. In our study, heritability was lowest for the youngest, highest for the middle, and -compared to the middle- slightly lower for the oldest cohort, which matches well with Steinberg & Morris’ [65] argumentation. However, since cohort differences could be due to variance differences between the cohorts, since our study was not longitudinal, and since the age difference between our cohorts is relatively large we cannot clearly support this view. Research focused on the specific traits underpinning EXT found mixed developmental patterns, with increasing genetic influence for some traits and no change in genetic influence for others. For example, the heritability of rule-breaking has been found to strikingly increase with pubertal development, whereas heritability of aggressive behavior showed no developmental differences [66]. A more detailed examination of the different traits of EXT may show more consistent trends and resolve some of the incoherent findings.

All in all, similar reasons may contribute to both the lack of differences between the cohorts for INT and the inconsistent pattern for EXT: the relatively old sample, different critical points of time for a change in the pattern, and different effects of the changing gene–environment interaction on both constructs. The relatively old sample in our study also does not allow us to generalize these results to children under the age of 10. Further research should address these issues by including younger children into the NTFD (although this brings methodological problems concerning self-reports), and longitudinal studies should consider the transition to adulthood—and thereby closing the gap between childhood and adult research—to test our suggestions.

### Limitations and further directions

Although the NTFD addresses several issues of the CTM, the current study has a few limitations which need to be mentioned. First, even with the inclusion of parents and siblings, it is still not possible to estimate non-additive genetic influences and sibling-specific environmental influences in the same model. To estimate both parameters simultaneously, data from offspring and partners of twins would be necessary, which, however, is rarely available. Second, with the univariate NTFD, only the passive gene–environment correlation can be estimated, but no other types of gene–environment interplay. Further research should examine different forms of gene–environment interplay in longitudinal designs, including measured environmental characteristics. Third, possible effects of sex were not explicitly analyzed in the current study. Due to the relatively small number of siblings and fathers our sample size was not sufficient to test sex-specific models. But, as sex was regressed out of the variables before running the NTFD, and since overall, previous research tended to find no or only negligible evidence for sex differences in heritability of INT and EXT [17, 50], it can be assumed that our results are valid for both sexes. Nevertheless, given the persistent interest in sex effects in this field, further research should consider sex differences in heritability of INT and EXT whenever possible to support this assumption. Fourth, compared to other-reports, self-reports may work better for INT than for EXT since INT symptoms are less easily observable for other raters while EXT symptoms are more noticeable and additionally often accompanied by a lower self-awareness and an under-reporting of the own symptoms [67]. Therefore, the use of self-reports is an advantage for INT but could be less beneficial in case of existing EXT symptoms. Though, given our representative and non-clinical sample, the impact of such a rater bias for EXT can be considered negligible. However, further work should consider additional information from other raters and clinical assessment. Finally, an actual strength of the current study which, however, has implications for the current research question needs to be addressed: We analyzed self-reports from an unselected population-based normative sample, resulting in low means for INT and EXT and a low prevalence of clinically relevant scores. Thus, the extent to which the pattern of genetic and environmental influences found in this study is representative for clinical or high-risk samples remains unclear. However, several studies on problem behavior have suggested that individuals with normal and pathological behavior differ in degree rather than in kind of behavior. This supported the view that the same sources affect normal and psychopathological variation in children and adolescents [64]. Therefore, the representativeness of our sample is beneficial and it is most likely that our results are generalizable to clinical samples, too.

## Conclusion

Our study provided a sophisticated and less biased disentanglement of genetic and environmental factors that underpin behavioral problems in childhood and adolescence compared to previous CTM-based work. By means of the NTFD, we separated effects of assortative mating and passive gene–environment correlation from different forms of shared environmental effects with simultaneous consideration of non-additive genetic effects. Compared to prior work, we showed that usually, heritability estimates obtained via CTM are slightly overestimated and therefore environmental influences are more important than presumed before. Besides this generally increased noticeability of the environment, finding shared environmental influences for INT significantly enhances the understanding of developmental outcomes. It also has implications for the development of interventions, because shared environmental influences are often relatively persistent and systematic, whereas non-shared influences are unsystematic and difficult to identify. Identifying these shared influences as solely twin-specific suggests that previous research may have misinterpreted shared environment to be family-specific, which supports existing doubts on the generalizability of shared environmental estimates obtained via CTM-based studies. Additionally, this finding underpins the importance of exclusively age-specific experiences in older children and adolescents. Further research has to identify these twin-specific environmental factors and to differentiate them from shared environmental factors on a family or non-twin sibling level to allow more specific treatment possibilities. Finding higher heritability and only non-shared environmental influences for EXT suggests that family similarities are mainly explained by genetic effects. Therefore, interventions might need to be less specific to living conditions shared within a family and could have greater success if they are oriented towards the broader environment.

Taken together, our results indicate that family-specific environment either contributes to behavior problems only on an individual level (meaning that every child perceives the same family environment differently) or that it has less influence than originally thought. When planning therapeutic interventions to counteract the genetic vulnerability of developing problem behavior, this must be considered. Additionally, as a result of correcting previously reported heritability estimates gained via twin studies downwards, we could explain a part of the gap between DNA-based heritability and twin studies. Finding lower heritability estimates in our self-report-based study, compared to earlier work with other raters, may also suggest that different genes influence measures of different informants, which would have implications for the phenotype definition in molecular genetic research [13]. All in all, more multivariate and longitudinal genetic research considering the whole family is needed to disentangle the complex interplay between genes and environment, to better understand the etiology of behavioral problems and to build the background for therapeutic interventions.

## Supporting information

S1 File Participant consent. (PDF)

S1 Table Measurement invariance. (PDF)

S2 Table Model parameters and variance components. (PDF)

S3 Table Model fit and standardized variance components if variances of children are standardized per cohort. (PDF)
