# Supplementary material for: On the etiology of internalizing and externalizing problem behavior: A twin-family study
Source: PLoS One. 2020 Mar 23;15(3):e0230626. doi: 10.1371/journal.pone.0230626 (PMC7089526; doi:10.1371/journal.pone.0230626)
Supplement: S1 File — (PDF) [file pone.0230626.s001.pdf]

Participant consent

The TwinLife study was conducted by the Social Research Institute TNS Infratest. TNS Infratest has a staff of about 900 well-trained interviewers distributed throughout Germany. All participants were informed in written form about the aims of the TwinLife study, the incentives and contact details. In addition they received a two-page leaflet summarizing the procedures concerning privacy and their rights to demand the deletion of all data collected in accordance with German data integrity laws. The written material was sent to participants prior to the interview. All of these documents were also available to the interviewers to be used again, if necessary, during the contact phase. All participants above the age of 14 were asked by the interviewer whether they consent with these regulations. If participants were under the legal age of 18 years, their parents were also asked for their consent. Participant's consent was recorded by the interviewer in the interview protocol. This procedure is in line with the German law and was reported to the ethics committee. It was chosen because TNS Infratest strongly recommended it, based on the concern that collecting actual signatures could result in a biased sample. Further information on the data collection procedures can be found in Brix et al. [1].

Reference

1. Brix, J., Pupeter, M., Rysina, A., Steinacker, G., Schneekloth, U., Baier, T., Gottschling, J., et al. (2017). *A longitudinal twin family study of the life course and individual development (TWINLIFE): Data collection and instruments of wave 1 face-to-face interviews* (TwinLife Technical Report Series, 05). Bielefeld: Project TwinLife "Genetic and social causes of life chances" (Universität Bielefeld / Universität des Saarlandes).
