# Supplementary material for: On the etiology of internalizing and externalizing problem behavior: A twin-family study
Source: PLoS One. 2020 Mar 23;15(3):e0230626. doi: 10.1371/journal.pone.0230626 (PMC7089526; doi:10.1371/journal.pone.0230626)
Supplement: S1 Table — (PDF) [file pone.0230626.s002.pdf]

S2 Table. Measurement invariance.

Model	χ^2/df (p)	CFI	RMSEA	AIC	X^2_{diff} / df_{diff} (p_{diff})
**Internalizing**					
M1. unconstrained (configural MI)	172,110 / 75 (.000)	.975	.020	412,110	--
M2. weights (metric MI)	303,292 / 109 (.000)	.950	.024	475,292	131,182 / 34 (.000)
M2.1. weights C11 = C17	247,173 / 92 (.000)	.960	.023	453,173	75,063 / 17 (.000)
M2.2. weights C11 = C23	250,444 / 92 (.000)	.960	.023	454,444	78,334 / 17 (.000)
M2.3. weights C17 = C23	230,896 / 92 (.000)	.964	.022	436,896	58,786 / 17 (.000)
M3. intercepts	715,382 / 129 (.000)	.850	.038	847,382	543,272 / 54 (.000)
M4. structural covariances	753,796 / 135 (.000)	.841	.039	873,796	581,686 / 60 (.000)
M5. Measurement residuals	1135,634 / 155 (.000)	.749	.045	1215,634	963,524 / 60 (.000)
**Externalizing**					
M1. unconstrained (configural MI)	180,621 / 54 (.000)	.957	.028	396,621	--
M2. weights (metric MI)	262,100 / 84 (.000)	.939	.026	418,100	81,479 / 30 (.000)
M2.1. weights C11 = C17	240,239 / 69 (.000)	.941	.028	426,239	59,618 / 15 (.000)
M2.2. weights C11 = C23	219,686 / 69 (.000)	.948	.027	405,686	39,065 / 15 (.001)
M2.3. weights C17 = C23	196,862 / 69 (.000)	.956	.025	382,862	16,241 / 15 (.366)
M3. intercepts	467,344 / 102 (.000)	.875	.034	587,344	286,723 / 48 (.000)
M4. structural covariances	537,600 / 108 (.000)	.853	.036	645,644	356,979 / 54 (.000)
M5. Measurement residuals	1239,495 / 126 (.000)	.619	.054	1311,495	1058,874 / 72 (.000)

M, Model; MI, measurement invariance; C, birth cohort; separate bifactor models of internalizing and externalizing problem behavior were estimated
