# Supplementary material for: On the etiology of internalizing and externalizing problem behavior: A twin-family study
Source: PLoS One. 2020 Mar 23;15(3):e0230626. doi: 10.1371/journal.pone.0230626 (PMC7089526; doi:10.1371/journal.pone.0230626)
Supplement: S2 Table — (PDF) [file pone.0230626.s003.pdf]

S3 Table. Model parameters and variance components.

	Model parameters								Standardized variance components						
Models	a	i	μ	m	f	ct	cs	e	a^2	i^2	$a^2m(1+\mu) + a^2f(1+\mu)$	$m^2+f^2+2mf\mu$	ct^2	cs^2	e^2
**Internalizing**															
cs = 0 (C11)	**.50**	-.10	**.14**	.01	-.02	**.42**		**.75**	.25	.01	.00	.00	.17		.57
cs = 0 (C17)	**.63**	.00	**.15**	.01	-.05	.16		**.77**	.39	.00	-.02	.00	.03		.59
cs = 0 (C23)	**.58**	-.21	**.18**	-.01	.01	-.30*		**.73**	.33	.05	.00	.00	.09		.53
i = 0 (C11)	**-.51**		**.14**	.01	-.03	**.42**	.00	**.75**	.26		-.01	.00	.17	.00	.57
i = 0 (C17)	**-.62**		**.15**	.01	-.05	.16	.06	**.77**	.39		-.01	.00	.03	.00	.60
i = 0 (C23)	**-.63**		**.18**	-.03	-.01	**.29**	.00	**.73**	.40		-.02	.00	.09	.00	.54
cs = 0, o. eq.	**.58**	-.11	**.15**	.00	-.03	**-.32**		**.75**	.33	.01	-.01	.00	.10		.56
cs = m = f = i = 0, o. eq.	**-.56**		**.15**			**.34**		**.75**	.32				.12		.57
**Externalizing**															
cs = 0 (C11)	**.48**	-.30*	.05	.00	-.03	-.17		**.81**	.23	.09	-.01	.00	.03		.66
cs = 0 (C17)	**.49**	**-.38**	**.17**	-.01	-.04	.00		**.79**	.24	.15	-.01	.00	.00		.62
cs = 0 (C23)	**.48**	**-.35**	**.17**	.01	.01	-.10		**.80**	.23	.12	.01	.00	.01		.63
i = 0 (C11)	**-.56**		.05	-.04	-.07	.15	.00	**.83**	.31		-.03	.01	.02	.00	.69
i = 0 (C17)	**-.62**		**.17**	-.06	-.09	.00	.00	**.81**	.38		-.07	.01	.00	.00	.67
i = 0 (C23)	**-.60**		**.17**	-.05	-.04	.00	.00	**.81**	.37		-.04	.00	.00	.00	.66
cs = 0, o. eq.	**.50**	**-.35**	**.12**	-.01	-.03	.00		**.80**	.25	.12	-.01	.00	.00		.64
cs = m = f = ct = 0 (C11)	**.46**	**-.36**	.05					**.81**	.21	.13					.66
cs = m = f = ct = 0 (C17)	**.45**	**-.42**	**.17**					**.79**	.20	.18					.62
cs = m = f = ct = 0 (C23)	**.50**	**-.34**	**.17**					**.80**	.25	.11					.63

C, birth cohort; o. eq: all other parameters were set equal across cohorts; a, additive genetic effects; i, non-additive genetic effects; μ, phenotypic spousal similarity; m, environmental transmission from mother to offspring; f, environmental transmission from father to offspring; ct, twin-specific shared environmental effects; cs, sibling-specific shared environmental effects; e, non-shared environmental effects (including measurement error); **bold**, $p < .001$; * $p < .01$
