# Supplementary material for: On the etiology of internalizing and externalizing problem behavior: A twin-family study
Source: PLoS One. 2020 Mar 23;15(3):e0230626. doi: 10.1371/journal.pone.0230626 (PMC7089526; doi:10.1371/journal.pone.0230626)
Supplement: S3 Table — (PDF) [file pone.0230626.s004.pdf]

S4.1 Table. Model fit if variances of children are standardized per cohort.

Model	χ^2 / df (p)	CFI	RMSEA	AIC	χ^2_{diff} / df_{diff} (p_{diff})
Internalizing					
**M1.1** (baseline)<br>cs = 0	54,20 / 69<br>(.904)	>.999	<.001	156,202	
**M2**<br>cs = 0, o. eq.	66,83 / 83<br>(.914)	>.999	<.001	140,827	12,63 / 14<br>(.556)
**M3 (final model)**<br>cs = i = m = f = 0; o. eq	68,76 / 86<br>(.913)	>.999	<.001	136,764	14,56 / 17<br>(.627)
Externalizing					
**M1.1** (baseline)<br>cs = 0	59,63 / 69<br>(.782)	>.999	<.001	161,622	
**M2**<br>cs = 0, o. eq.	67,36 / 83<br>(.894)	>.999	<.001	141,358	7,73 / 14<br>(.903)
**M3 (final model)**<br>cs = m = f = ct = 0; o. eq.	68,79 / 86<br>(.913)	>.999	<.001	136,786	9,16 / 17<br>(.935)

M, Model; C, birth cohort; cs, environmental effects shared by siblings; i, non-additive genetic effects; m, environmental transmission from mother to offspring; f, environmental transmission from father to offspring; ct, environmental influences shared by twins; o. eq., all other Parameters were set equal across cohorts

S4.2 Table. Standardized variance components if variances of children are standardized per cohort (final models).

	a^2	i^2	ct^2	e^2
Internalizing	.32<br>[.302 – .330]		.11<br>[.094 - .134]	.57<br>[.563 - .577]
Externalizing	.22<br>[.204 - .234]	.14<br>[.120 - .170]		.64<br>[.628 - .645]

C, birth cohort; [], 95% confidence interval; a, additive genetic effects; i, non-additive genetic effects; ct, twin-specific shared environmental effects; e, non-shared environmental effects (including measurement error)
